# Automated assessment of paraspinal muscle fat composition based on the segmentation of chemical shift encoding-based water/fat-separated images

**DOI:** 10.1186/s41747-018-0065-2

**Published:** 2018-11-07

**Authors:** Thomas Baum, Cristian Lorenz, Christian Buerger, Friedemann Freitag, Michael Dieckmeyer, Holger Eggers, Claus Zimmer, Dimitrios C. Karampinos, Jan S. Kirschke

**Affiliations:** 1 Department of Diagnostic and Interventional Neuroradiology, Klinikum rechts der Isar, Technical University of Munich, Ismaninger Str. 22, 81675 Munich, Germany; 2 Philips Research Laboratories, Hamburg, Germany; 3 Department of Diagnostic and Interventional Radiology, Klinikum rechts der Isar, Technical University of Munich, Munich, Germany

**Keywords:** Biomarkers, Magnetic resonance imaging, Paraspinal muscles, Proton-density fat fraction, Sarcopenia

## Abstract

Proton-density fat fraction (PDFF) of the paraspinal muscles, derived from chemical shift encoding-based water-fat magnetic resonance imaging, has emerged as an important surrogate biomarker in individuals with intervertebral disc disease, osteoporosis, sarcopenia and neuromuscular disorders. However, quantification of paraspinal muscle PDFF is currently limited in clinical routine due to the required time-consuming manual segmentation procedure. The present study aimed to develop an automatic segmentation algorithm of the lumbar paraspinal muscles based on water-fat sequences and compare the performance of this algorithm to ground truth data based on manual segmentation. The algorithm comprised an average shape model, a dual feature model, associating each surface point with a fat and water image appearance feature, and a detection model. Right and left psoas, quadratus lumborum and erector spinae muscles were automatically segmented. Dice coefficients averaged over all six muscle compartments amounted to 0.83 (range 0.75–0.90).

**Electronic supplementary material:**

The online version of this article (10.1186/s41747-018-0065-2) contains supplementary material, which is available to authorized users.

## Key points

- An MRI-based automatic segmentation algorithm of the lumbar paraspinal muscles was developed.
- Paraspinal muscles were automatically segmented with an averaged Dice coefficient of 0.83.
- The algorithm may support the clinical application of proton-density fat fraction as imaging biomarker.

## Background

Magnetic resonance imaging (MRI)-based assessment of the fat composition of the paraspinal muscles has been proposed as a surrogate marker in individuals with intervertebral disc disease, osteoporosis, sarcopenia, and neuromuscular disorders [1, 2]. Using chemical shift encoding-based water-fat MRI, the proton-density fat fraction (PDFF) of each paraspinal muscle compartment could be reliably extracted [3, 4]. In clinical routine, water-fat MRI-based assessment of paraspinal muscle PDFF is currently limited due to the time-consuming manual segmentation procedure.

Gawel et al. [5] introduced a method for automatic segmentation of vertebral column tissue based on machine learning with cascade classifiers, active appearance model and principal component analysis. Further approaches have been reported for automatic localisation and segmentation of vertebral bodies on MRI. For instance, Chu et al. [6] used a random forest regression and classification framework and Hille et al. [7] used computed appearance-based vertebral body probability maps with a subsequent hybrid level-set segmentation. Available segmentation methods have been summarised in a review by Rak et al. [8].

However, little research is available on automatic segmentation of paraspinal muscles on MRI. Engstrom et al. [9] used statistical shape modelling for the segmentation of the quadratus lumborum muscle in T1-weighted images. Jurcak et al. [10] applied a hybrid atlas-based geodesic active contour algorithm for the automated segmentation of the quadratus lumborum muscle.

Therefore, the purpose of this study was to develop an automatic segmentation algorithm of the paraspinal muscles relying on chemical shift encoding-based water-fat MRI and compare the performance of this algorithm to ground truth data based on manual segmentation.

## Methods

### Participants

The study was approved by the local institutional committee for human research and in accordance with the 1964 Helsinki declaration and its later amendments. All individuals gave written informed consent before participation in the study.

Ten healthy individuals were recruited for this study (eight men and two women, age 29 ± 8 years [mean ± standard deviation (SD)], and body mass index 26.7 ± 2.3 kg/m^2^ [mean ± SD]).

### MRI protocol

All participants underwent MRI at two time points (baseline and six-week follow-up) to obtain longitudinal imaging data for long-term reproducibility purposes. The lumbar musculature of the individual was scanned on a 3-T whole-body scanner (Ingenia, Philips Healthcare, Best, The Netherlands) using the built-in-the-table posterior coil elements (12-channel array). An axially prescribed six-echo three-dimensional spoiled gradient-echo sequence was used for chemical shift encoding-based water-fat separation. The sequence acquired the six echoes in a single time of repetition using non-flyback (bipolar) read-out gradients and the following imaging parameters: time of repetition 11 ms; minimum time of echo 1.04 ms; ΔTE 0.8 ms; field of view 220 × 220 × 219 mm; acquisition matrix 72 × 110 × 73; acquisition voxel size 3.1 × 2.0 × 3.0 mm; frequency encoding direction left to right; receiver bandwidth 2756 Hz/pixel; scan time 2:01 min. A flip angle of 3° was used to minimise T1-bias effects [11].

### Image-based fat quantification

The gradient-echo imaging data were processed on-line using the mDIXON Quant software provided by the manufacturer. It performs a complex-based water-fat decomposition using a pre-calibrated seven-peak fat spectrum and a single T2* to model the signal variation with echo time. PDFF maps were then computed as the ratio of the fat signal over the sum of fat and water signals.

### Manual segmentation

**Fig. 1:**
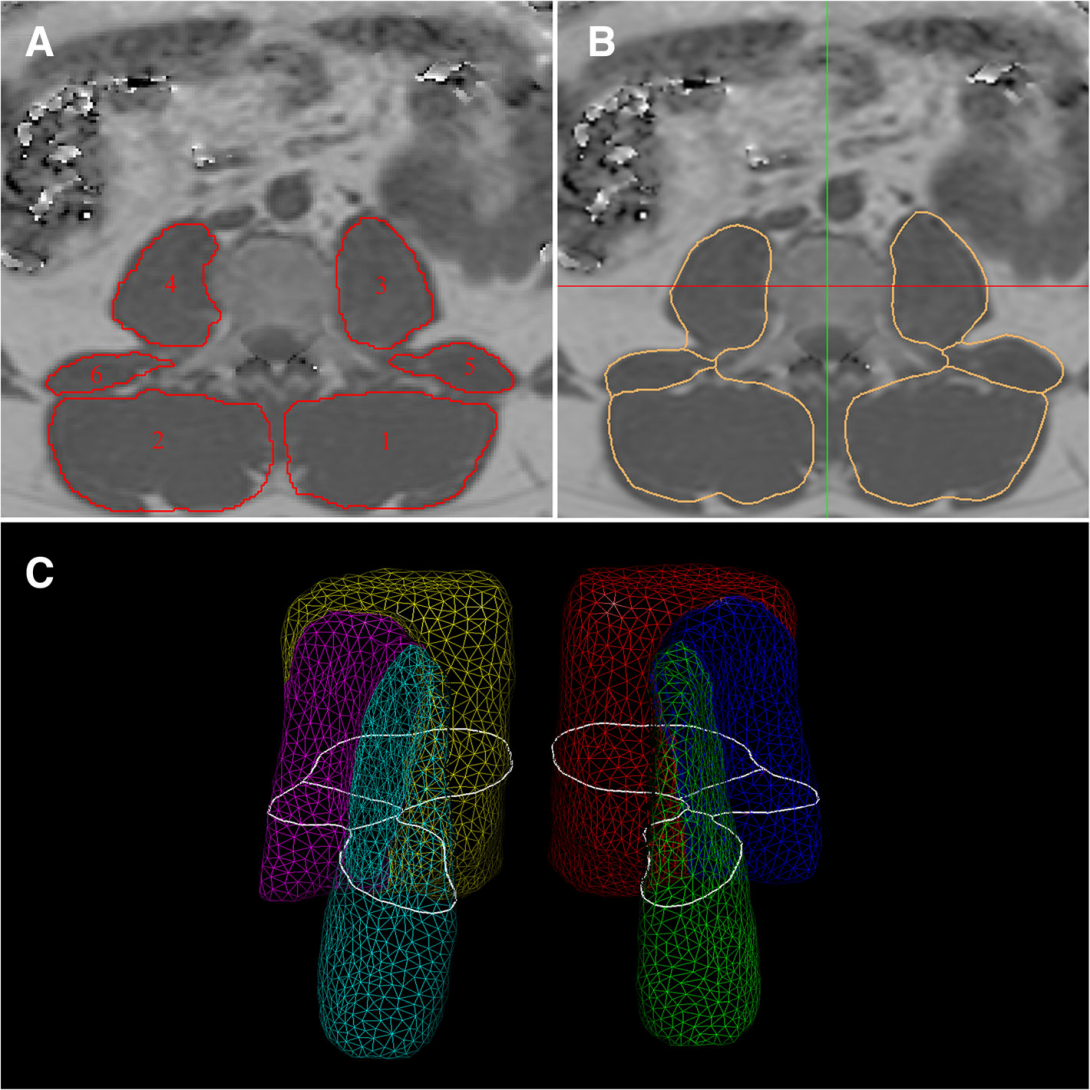
Representative PDFF *maps*. **a** Manually segmented muscle compartments as ground truth: 1, left erector spinae muscle; 2, right erector spine muscle; 3, left psoas muscle; 4, right psoas muscle; 5, left quadratus lumborum muscle; 6, right quadratus lumborum muscle. **b** Results of the automatic segmentation of the muscle compartments. **c** Average triangular surface model with cross-sectional cut-contour of central axial slice depicted in *white*

Manual segmentation of the paraspinal muscles was performed on the PDFF maps at baseline and follow-up by using the free open-source software Medical Imaging Interaction Toolkit (MITK), developed by the Division of Medical and Biological Informatics, German Cancer Research Center, Heidelberg, Germany (www.mitk.org). The following six muscle compartments were separately segmented by one operator from the upper endplate level of L2 to the lower endplate level of L5: right and left psoas muscles; right and left quadratus lumborum muscles; and right and left erector spinae muscles (Fig. 1a).

### Automatic segmentation

Baseline and follow-up images and corresponding manual muscle segmentations of seven individuals were used as a training dataset and those of the remaining three individuals as a test dataset. Manual muscle segmentations in the baseline and follow-up images of the three test participants served as ground truth and were considered as gold standard for the automatic muscle segmentation results. Based on the manual segmentations of the training set, a model of the six muscle compartments was generated. It comprised an average-shaped model, represented as triangle mesh, a dual-feature model, associating each surface triangle with a fat and water image appearance feature, and a detection model [12]. For its generation, first a shape model was created. A fuzzy averaging approach as described in Blaffert et al. [13] was followed to convert the label images resulting from the manual segmentation step to an average multi-compartment surface model. Second, a feature model that relates surface positions with corresponding image features, such as intensity edges, was generated following Peters et al. [14]. To do so, a training set of images with corresponding mesh models was created by adapting the average-shaped model from the previous step to the manual segmentation results. In this step, a simplified version of the model-based segmentation method [15] was applied, using a simple gradient feature to adapt the average-shaped model to the manually created label images. During the training phase, optimal local features are determined for the fat as well as the water image.

For the automatic segmentation of an unseen image, first a generalised Hough transform for structure localisation is performed to initialise the model in the patient image, followed by a coarse-to-fine individualisation of the surface model [15, 16]. During individualisation, an objective function consisting of an image feature match term and a shape deviation term was evaluated for optimising pose and shape of the model. Two image features, for water and fat image, were evaluated simultaneously. The procedure was iterated, allowing first only a rigid transformation of the model and later a free-form deformation to obtain a detailed muscle delineation (Fig. 1b and c; Additional file 1).

### Statistical analysis

Dice coefficients [17] were determined to compare the automatic muscle segmentations with the corresponding ground truth. Wilcoxon signed rank tests were used to assess differences of muscle volume and PDFF based on automatic segmentation and ground truth, respectively.

## Results

The Dice coefficient averaged over all six muscle compartments amounted to 0.83 (range 0.75–0.90). The highest Dice coefficients were observed for the erector spinae muscles (right 0.89, left 0.90), followed by the psoas muscles (right 0.83, left 0.77). The lowest Dice coefficients were found for the quadratus lumborum muscles (right 0.75, left 0.76).

Mean volume and PDFF of each muscle compartment for the training dataset are listed in Table 1. The automatic segmentation algorithm significantly overestimated the muscle volumes of right (*p* = 0.012) and left (*p* = 0.012) erector spinae and right (*p* = 0.025) and left (*p* = 0.017) psoas muscles. Absolute differences in PDFF values obtained with the automatic and the manual muscle segmentation were relatively small (range 0.02–0.58%), but statistically significant (*p* < 0.012) in the erector spinae muscles (Table 1).

**Table 1:** Mean and standard deviation of PDFF (%) and volume (cm^3^) of each muscle compartment in the training and test dataset

	Training set (*n* = 14)		Testing set (*n* = 6)						
	Fat fraction	Volume	GT fat fraction	AS fat fraction	Δ fat fraction	*p*	GT volume	AS volume	*p*
Right erector spinae	6.66 ± 2.07	241 ± 56	5.10 ± 1.09	5.49 ± 1.14	0.39	0.012	235 ± 60	287 ± 74	0.012
Left erector spinae	6.29 ± 2.19	252 ± 53	3.44 ± 1.88	4.02 ± 1.90	0.58	0.012	238 ± 56	293 ± 74	0.012
Right quadratus lumborum	6.44 ± 5.21	45 ± 10	5.36 ± 1.22	4.86 ± 0.72	0.50	0.025	43 ± 13	45 ± 14	0.263
Left quadratus lumborum	7.46 ± 5.82	51 ± 13	4.24 ± 3.27	3.69 ± 2.43	0.55	0.123	47 ± 14	47 ± 15	0.889
Right psoas	4.19 ± 2.65	121 ± 28	4.01 ± 1.68	3.82 ± 1.56	0.19	0.093	117 ± 35	130 ± 45	0.025
Left psoas	4.04 ± 3.87	119 ± 33	2.98 ± 2.58	2.96 ± 2.22	0.02	0.575	109 ± 35	135 ± 51	0.017

*p* values refer to the comparison of automatic segmentation (AS) and ground truth (GT) results in the test dataset

## Discussion

The proposed algorithm for automatic paraspinal muscle segmentation on chemical shift encoding-based water-fat MRI showed small absolute errors in PDFF (range 0.02–0.58%) in the scanned healthy participants.

The Dice coefficients observed in our study were comparable to those reported by Jurcak et al. [10]. They applied a hybrid atlas-based geodesic active contour algorithm for the automated segmentation of the quadratus lumborum muscle and obtained Dice coefficients for the right and left quadratus lumborum muscles of 0.78 and 0.75, respectively. Similarly, Engstrom et al. [9] reported Dice coefficients of 0.87 for automated segmentation of the quadratus lumborum muscles based on statistical shape modelling. Differences in PDFF values between the automatic segmentation and ground truth were relatively small and clinically acceptable (absolute difference range from 0.02% to 0.58%). In the future, machine learning methods may be an alternative approach for the segmentation of the paraspinal muscles in water-fat MR images as previously applied for the segmentation of the vertebral column tissues [5].

PDFF in the erector spinae muscles and the volumes of erector spinae and psoas muscle were significantly greater by using the automated segmentation algorithm compared to the manually segmented ground truth. These findings may result from the different segmentation approaches. The operator tried to avoid the accidental inclusion of epimuscular fat and placed the regions of interest (ROI) within in the inner contour of the visible muscle boundaries. The automated segmentation algorithm detected the muscle boundaries and exactly placed the ROIs on the muscle boundaries. In the future, a circular shrinking of the automatically placed ROIs can be implemented to reduce the volume differences between automated and manual segmentation.

To further improve our existing algorithm, an increased number of participants for the training dataset is necessary to reliably extract muscle volume and PDFF of the erector spinae muscles, especially when applying the method in atrophic muscles affected by pathology.

In conclusion, an automatic segmentation algorithm of the lumbar paraspinal muscles was developed and an averaged Dice coefficient of 0.83 was obtained between automated segmentations and manually segmented ground truth.

## Additional file

Additional file 1: Representative fat images with automated segmentation results of the six muscle compartments (red contours). (MOV 1036 kb)

## Abbreviations

MRI: Magnetic resonance imaging
PDFF: Proton-density fat fraction
ROI: Region of interest
